# Loneliness and mental burden among German medical students during the fading COVID-19 pandemic: a mixed-methods approach

**DOI:** 10.3389/fpsyg.2025.1526960

**Published:** 2025-03-25

**Authors:** Marvik Leich, Jennifer Guse, Corinna Bergelt

**Affiliations:** ^1^Department of Medical Psychology, University Medicine Greifswald, Greifswald, Germany; ^2^Department of Medical Psychology, University Medical Center Hamburg-Eppendorf, Hamburg, Germany

**Keywords:** medical students, COVID-19 pandemic, loneliness, mental health, undergraduate medical education

## Abstract

**Introduction:**

Medical students experience significantly more mental burdens compared to the general public. This circumstance was further exacerbated by the pandemic, particularly with regard to loneliness. While previous studies have identified risk factors for loneliness among students of different subjects, recent data focusing on medical students during the late stages of the pandemic remain insufficient. This study aims to address this gap.

**Methods:**

We performed a cross-sectional study at a German Medical School, consisting of two online surveys conducted in winter 2021/22 and summer 2022. The study sample, composed of 283 undergraduate students in winter 2021/22 (231 in summer 2022), answered five well-established scales for measuring loneliness (R-UCLA3), distress (DT-NCCN), anxiety (GAD-2), depression (PHQ-2), and perceived stress (PPS-4). Additionally, we evaluated burdensome aspects of students' current situation through qualitative analysis. Longitudinal analyses were conducted for 80 medical students who participated in both surveys.

**Results:**

Around 20% of all students responded to each survey. In winter 2021/22, 55.1% of students reported loneliness above the cut-off (*M* = 5.77 [2.02]; decreasing to 45.0% by summer 2022 (*M* = 5.22 [1.90]). Lonely students reported significantly higher levels of depression, anxiety, and self-perceived stress in both survey periods. Overall distress increased substantially throughout our study (Cohen's *d* = −0.54). Binary regression models indicated a shift in loneliness risk factors: in winter 2021/22, being single, higher self-perceived stress levels, and decreased study motivation were associated with increased loneliness. Lower peer connectedness emerged as the sole significant factor associated with loneliness in summer 2022. While the pandemic-related burden on students' study motivation lessened, issues related to exam preparation and lack of study organization through the faculty increased, varying significantly depending on students' study year.

**Conclusion:**

Our data suggest that loneliness among German medical students decreased in the late stages of the COVID-19 pandemic. However, other mental burdens persisted at high levels compared to other data in the general public and medical students. Students' responses underscore the need for improved academic support by eased study program structure, improved counseling, and tailored services for students of different study years.

## 1 Introduction

SARS-CoV-2 caused tremendous disruption to global health policy, profoundly affecting all aspects of social life. Within the SARS-CoV-2 pandemic, almost 800 million cases were reported (World Health Organization, 2024). Whereas initial research has mainly focused on the elderly populations who face a greater risk of severity and mortality after a COVID-19 infection (Zhang et al., 2023), research has neglected the impact of the pandemic on the psychological well-being of adolescents and young adults for a long time. Even before the pandemic, young adults were vulnerable to mental health concerns (Gulliver et al., 2010; Gustavson et al., 2018; Health NIoM, 2023), and had the highest prevalence of mental illness compared to other adults (Gustavson et al., 2018; Health NIoM, 2023). As a vulnerable group, young adults have been especially impacted by a mental health burden through the pandemic, including a rise in levels of depression, anxiety, and loneliness (Jones et al., 2021; Meherali et al., 2021; Oliveira et al., 2022). Due to pandemic-related policies, the educational sector, which plays a crucial role in young adults' daily lives, was especially struck, leading to learning losses in tertiary education learners (Tang, 2023), especially medical students. COVID-19 particularly impacted medical students, as medical education was unprecedentedly altered because of university hospitals' crucial role as healthcare providers and research facilities, leading to suspensions of clinical rotations and interruption of medical licensing exams (Tang, 2023; Bundesgesundheitsministerium, 2020). Unparalleled, medical students faced unprecedented stress factors in their dual role as students, and, in many cases, as healthcare workers combating COVID-19. As a result, COVID-19 directly influenced medical students' perception of future workplaces, leading to uncertainties about future residencies (Tang, 2023), and career plans (Dedeilia et al., 2023; Alrumi, 2024). After all, international research conducted in early 2020 underscored the outstanding negative impacts on medical students' mental health caused by the COVID-19 pandemic (Tahir et al., 2021; Halperin et al., 2021; Guse et al., 2021a). However, mental health concerns among medical students have not been a new uprising problem since COVID-19. More than one decade ago, studies described a high mental burden among medical students due to depression and anxiety (Wolf et al., 1998; Tjia et al., 2005). Moreover, medical students exhibited more frequent symptoms of depression and suicidal ideas (Rotenstein et al., 2016), distress (Dyrbye et al., 2006), burnout (Dyrbye et al., 2014), alcohol abuse/dependence (Jackson et al., 2016), poorer sleep quality, and excessive daytime sleepiness (Jahrami et al., 2020) than the general population.

A particularly relevant concern of mental burden emerges with loneliness, whose adverse effects are comprehensive. For one thing, loneliness predicts higher blood pressure and morning rises in cortisol, which are linked to reduced immunocompetence (Hawkley et al., 2006). Further, loneliness worsens cognitive performance in executive function (Cacioppo and Hawkley, 2009). More compelling, a lack of good and strong relationships (Holt-Lunstad et al., 2010), social isolation, and loneliness (Holt-Lunstad et al., 2015) are linked to higher odds of mortality, even surpassing risk factors such as obesity and reduced physical activity. Social isolation seemingly plays a key role in developing burnout among medical students (Gradiski et al., 2022). Similar to other mental burdens, loneliness became a growing mental health concern among young adults before the pandemic (Hysing et al., 2020). With the onset of COVID-19 in 2020, loneliness levels increased in the general population (Bu et al., 2020a,b); this effect is supported by systematic review and meta-analysis (Ernst et al., 2022). Levels of loneliness likely increased in medical students worldwide (Alkureishi et al., 2022; ElHawary et al., 2021), as well as in Germany (Werner et al., 2021; Weber et al., 2022).

Previous research among students provided substantial evidence on risk factors and factors associated with loneliness, such as depression (Werner et al., 2021) and anxiety (Diao et al., 2023), female gender (Hysing et al., 2020; Werner et al., 2021; Labrague et al., 2021) being single (Hysing et al., 2020; Werner et al., 2021), living alone (Hysing et al., 2020; Diehl et al., 2018), being young (Hysing et al., 2020; Diehl et al., 2018) or old (Hysing et al., 2020), year of study (Dagnew and Dagne, 2019) and various social and interpersonal variables (social support, peer relationships, and life events) (Zhang et al., 2021). As most of this data was collected prior to the pandemic or in the first year of COVID-19, little is known about how the phenomenon of loneliness has evolved toward the end of the pandemic. This study aims to investigate how the outgoing COVID-19 pandemic affects medical students' mental burden, focusing on loneliness and its possible predictors.

## 2 Materials and methods

### 2.1 Study design

The observational study consisted of two voluntary online surveys. It was performed at one medical school in Germany (Greifswald University Medical School, abbr. UMG) with the primary purpose of collecting information associated with loneliness and mental health of medical and dental students during the COVID-19 pandemic. Students were invited to participate at the end of the winter semester 2021/22 (February to March 2022, also described as survey t1 or “winter 2021/2022”) and the summer semester 2022 (July to September 2022, survey t2 or “summer 2022”). The highest peak in weekly SARS-CoV-2 infections in Germany was registered during the winter survey (World Health Organization, 2024).

Participants created pseudonyms only known to them at the beginning of each survey. Due to the pseudonyms, data of participants who answered at both time points (*n* = 80) could be connected, resulting in longitudinal observation and analyses.

### 2.2 Participants

All medical and dental students enrolled at UMG (1.344 at t1 and 1.314 at t2) were invited to participate in our exploratory study. Based on calculations by G^*^Power 3.1 (Faul et al., 2009), the achieved sample sizes (t1: *N* = 283, t2: *N* = 231) were sufficient enough to ensure robust statistical analyses, considering an alpha level of 0.05, a power of 95 %, and at least small effect sizes of the respective statistical tests [*t*-test: Cohen's *d* = 0.2, and chi-square test: phi = 0.3, (Cohen, 1988)]. Both surveys were promoted through e-mail by the Dean of Students Office and the Medical Students Council of UMG. In addition, invitations to participate were distributed via social media groups and posters at places of student life (central locker room, students' skills lab, and Dean's office). Initial invitations were sent to students at the beginning of the evaluation period, which takes place at the start of the lecture-free period in February and July. Reminders were sent during the remainder of the semester. Study participants did not receive any rewards or incentives for their contributions. Seven questionnaires in t1 and 16 in t2 were excluded during the data cleaning process due to duplicate pseudonyms, resulting from an attempt to participate two times in one survey. Two respondents were non-binary and excluded from the analyses since the anonymity of the respondents could not be guaranteed.

### 2.3 Measures

First, sociodemographic characteristics were gathered (gender, age, subject of studies, current semester of study, housing situation, and relationship status). Undergraduate medical studies in the medical school UMG are distributed over 6 years (12 semesters), with the first medical licensing examination after the second year. Clinical training at UMG starts in the third year. Loneliness, stress, anxiety, and depressiveness were assessed with validated scales. Established and self-constructed items assessed students' study situation and motivation. Finally, we included one open-ended question to assess burdensome aspects of the current study situation.

#### 2.3.1 Loneliness

Participants' loneliness was measured using the ultrashort 3-item version of the Revised UCLA Loneliness Scale (R-UCLA). It comprises three dimensions of loneliness: interpersonal connectedness, social connectedness, and self-perceived isolation (1 = hardly ever, 2 = sometimes, 3 = often). Categorization into “not lonely” (sum score 3–5) and “lonely” (sum score 6–9) has been carried out in the past. The scale demonstrates both satisfactory reliability and validity for the US population (Hughes et al., 2004), and has since been widely used worldwide. However, a German psychometric validation exists only for the 20-items revised UCLA Loneliness Scale (Döring, 1993).

#### 2.3.2 Distress

To measure the overall psychosocial stress, we used the German version of the Distress Thermometer (DT), a screening instrument initially developed by the National Comprehensive Cancer Network (NCCN) for cancer patients. It consists of a scale from 0 to 10, with higher scores indicating high distress levels. In the NCCN guidelines, 5- or a higher cut-off value is recommended for the German-speaking region to identify significantly distressed persons. The DT has proven to be a valid screening instrument for cancer populations (Mehnert et al., 2006). Similar to other widely used single-item scales (Buchberger et al., 2019; Elo et al., 2003), its brevity and good applicability outweigh its lack of psychometric validation in the German general population.

#### 2.3.3 Depression and anxiety

Depression and anxiety symptoms were assessed using the German version of the Patient Health Questionnaire (PHQ-4) (Lowe et al., 2010). The PHQ-4 is an ultra-short format of the Patient Health Questionnaire (PHQ-D), consisting of four questions from the modules depressiveness (PHQ-2) and generalized anxiety (GAD-2). The PHQ-4 measures self-perceived impairment due to depressive or anxiety symptoms over the last 2 weeks (0 = not at all, 3 = almost every day). Cut-off values above 2 points in the GAD-2 and PHQ-2 modules are indicative of clinically relevant levels of depressive and anxiety symptoms. To assess overall mental burden, the sum score of the PHQ-4 can be categorized into normal (0–2), mild (3–5), moderate (6–8), and severe (9–12) levels of distress. In the German population (Wicke et al., 2022), the screening instrument is tested to be highly valid and reliable.

#### 2.3.4 Self-perceived stress and study situation

Students' self-perceived stress was assessed with the four-item Perceived Stress Scale (PSS-4), a validated and recognized test instrument (Cohen, 1988; Klein et al., 2016). Referring to the last month, the PPS-4 assesses stressful experiences in 4 items on a five-point rating scale (0 = never, 4 = very often). Higher sum scores indicate higher stress levels. Further, one self-constructed item measured the self-perceived changes in study motivation (1 = considerably improved, 5 = considerably worsened).

One item related to the participants' learning environment was included from the John Hopkins Learning Environment Scale to assess students' community of peers (Shochet et al., 2015).

#### 2.3.5 Burdensome aspects

One open-ended question at the end of the surveys assessed burdensome aspects of students' current study situations (What are you most concerned about in your current study situation?).

### 2.4 Statistical analysis

#### 2.4.1 Quantitative data

The study population was characterized by descriptive analysis. Depending on the scale quality of the respective sociodemographic variables, group comparisons were carried out with chi-square tests, *T*-tests, and univariate analysis of variance (ANOVA). Holm-Bonferroni corrections were applied to correct false-positive results due to multiple testing (Holm, 1979). Binary logistic regressions were performed to investigate loneliness-related factors and whether factors changed during the fading pandemic. In addition to age, gender, and study subject, our models included variables that significantly differed between lonely and not lonely subjects in univariate analyses. Selected variables were entered simultaneously in the model without exclusion by insignificance or inadequate model fit (i.e., forced-entry method). All quantitative analyses were carried out with IBM SPSS, version 29 (IBM, 2023).

#### 2.4.2 Qualitative data

The one open-ended question was processed qualitatively by standards of conventional content analysis (Hsieh and Shannon, 2005). For this purpose, answers were inductively analyzed in terms of underlying concepts, thus identifying key topics. Then, the first higher-level category system was developed. Researchers JG and ML independently applied the first higher-level category system for test coding the open questions in our surveys and agreed on a final form of coding. Interrater reliability proved to be at least substantial (κ ≥ 0.60) in 70.4 % of all items, according to Landis and Koch (1977). Group comparisons were conducted using chi-square tests. Subsequent coding and analyses were done in IBM SPSS, version 29 (IBM, 2023).

## 3 Results

In total, 283 students participated in the winter survey (t1), representing 21.1% of all eligible students (t2: *N* = 231, 17.6%), 71.7 % (winter survey) and 71.4 % (summer survey) were female.

Most students were between 21 and 25 years old and had a relationship (t1: 56.2 %, t2: 52.4 %). The proportion of undergraduate students in the preclinical and clinical training was almost equal. The study samples, t1 and t2, did not differ significantly with regard to gender, age, study year, and study subject. Similarly, most sociodemographics of medical and dental students did not differ significantly except for age in t2 (Table 1).

**Table 1:** Students' characteristics.

	**First survey in winter 2021/22**				**Second survey in summer 2022**				**Longitudinal group**
	**Total**	**Medical**	**Dental**		**Total**	**Medical**	**Dental**		
		**Students**				**Students**			
	***n =* 283**	***n =* 256**	***n =* 27**	**χ^2^ (*p*)**	***n =* 231**	***n =* 207**	***n =* 24**	**χ^2^ (*p*)**	***n =* 80**
**Sex**	**%**	**%**	**%**		**%**	**%**	**%**		**%**
*Male*	28.3	28.9	22.2	0.538 (0.463)	28.6	28.5	29.2	0.005 (0.946)	28.7
*Female*	71.7	71.1	77.8		71.4	71.5	70.8		71.3
**Age**	***M** =* **23.3 (SD** = **3.2)**				***M** =* **23.8 (SD** = **3.4)**				***M** =* **22.9 (SD** = **3.2)**
≤ 20 years	19.1	19.5	14.8	3.021 (0.221)	13.4	13.5	12.5	0.275 (0.871)	25.0
21–25 years	59.0	57.4	74.1		61.9	61.4	66.7		57.5
≥26 years	21.9	23.1	11.1		24.7	25.1	20.8		17.5
**Study year**	***n***	**%**	**%**		***n***	**%**	**%**		***n***
Year 1	75	25.0	40.7	3.360 (0.339)	54	22.2	33.3	16.101 **(0.001)**	19
Year 2	67	23.8	22.2		58	28.0	00.0		28
Year 3	64	23.0	18.5		47	21.7	08.3		14
Year 4 +	77	28.1	18.5		72	28.0	58.3		19

*n* = frequencies, % = percentages. M = mean value, SD = standard deviation. χ^2^ = chi-square-value. Bold *p*-value = significant after Holm-Bonferroni correction. Differences between t1 and t2 regarding gender (χ^2^ = 0.006), age (χ^2^ = 3.062), and study year (χ^2^ = 1.594) are insignificant (*p*> 0.05).

### 3.1 Loneliness

55.1% of all students scored above the cut-off and were categorized as “lonely” in the winter survey (45.0% in the summer survey t2). Lonely students experienced significantly higher levels of distress in winter 2021/22 than their counterparts. Furthermore, they reported significantly higher levels of depressive symptoms, anxiety, and stress in both surveys, with computed effect sizes translating to moderate and almost large effects. This underscores the impact of loneliness on levels of mental burden in our study sample. While lonely students expressed significantly lower study motivation in the winter survey, this difference was no longer observable in the summer survey. However, lonely students expressed significantly lower means of connectedness to fellows in both measurements (Table 2). Generally, levels of mental burdens varied significantly among students based on their year of study, with students in their fourth study year and beyond globally reporting lower levels of mental burdens than academically younger fellows. Loneliness was the only exception to this trend, as differences in study years did not vary considerably (Supplementary Table S1) Amongst students who participated in both surveys, levels of loneliness decreased from winter to summer, revealing a tendency, even if not significant, due to Holm-Bonferroni correction. Similarly, levels of connectedness to fellows rose. General distress significantly increased from winter to summer, while depressive symptoms, anxiety, stress, and study motivation did not relevantly change (Table 3). Examination of the prevalence of loneliness by sociodemographic characteristics (age, gender, study subject, study year, living situation, and relationship status) revealed that students living alone reported loneliness significantly more frequently in the winter survey. The results from the summer survey showed no significant divergence in loneliness based on participants' sociodemographic characteristics. Regarding the prevalence of loneliness by mental burden, those students who exceeded the cut-off values of depression and anxiety reported significantly more often being lonely in both surveys. Effect sizes indicated weak to moderate associations. Globally speaking, data shows a rise in shares of feeling distressed (64.2%−66.7%), depressed (42.8%−51.9%), and anxious (44.9%−66.7%) in the course of our study (Table 4).

**Table 2:** Mental burden and loneliness in 514 medical students in the fading COVID-19 pandemic in 2022.

		**Total sample**	**Not lonely**	**Lonely**					
	**Winter 2021/22**	***n =* 283**	***n =* 127**	***n =* 156**					
	**Summer 2022**	***n =* 231**	***n =* 127**	***n =* 104**					
**Variables**	**Survey**	***M* (SD)**	***M* (SD)**	***M* (SD)**	**DM**	**SE**	***T*-value**	***p*-value**	**Cohen's *d***
**Distress**	Winter 2021/22	5.43 (2.65)	4.83 (2.76)	5.91 (2.47)	−1.07	0.31	−3.457	**< 0.001**	−0.41
	Summer 2022	6.40 (2.70)	6.09 (2.85)	6.82 (2.45)	−0.72	0.35	−2.042	0.042	−0.27
**Overall symptoms of depression and anxiety**	Winter 2021/22	4.98 (3.23)	3.76 (2.95)	5.97 (3.12)	−2.20	0.36	−6.054	**< 0.001**	−0.72
	Summer 2022	5.42 (3.33)	4.72 (3.28)	6.27 (3.19)	−1.55	0.43	−3.626	**< 0.001**	−0.48
**Depression**	Winter 2021/22	2.44 (1.71)	1.82 (1.55)	2.95 (1.67)	−1.13	0.19	−5.847	**< 0.001**	−0.70
	Summer 2022	2.64 (1.81)	2.28 (1.79)	3.07 (1.75)	−0.78	0.24	−3.342	**< 0.001**	−0.45
**Anxiety**	Winter 2021/22	2.54 (1.80)	1.84 (1.64)	3.02 (1.79)	−1.07	0.21	−5.213	**< 0.001**	−0.62
	Summer 2022	2.78 (1.79)	2.43 (1.74)	3.20 (1.76)	−0.77	0.23	−3.321	**0.001**	−0.44
**Self-perceived Stress**	Winter 2021/22	6.30 (3.63)	4.89 (3.42)	7.44 (3.39)	−2.55	0.40	−6.269	**< 0.001**	−0.75
	Summer 2022	6.42 (3.49)	5.65 (3.82)	7.36 (3.42)	−1.70	0.45	−3.789	**< 0.001**	−0.47
**Change in study motivation**	Winter 2021/22	3.51 (0.84)	3.28 (0.78)	3.71 (0.84)	−0.43	0.10	−4.420	**< 0.001**	−0.53
	Summer 2022	3.46 (0.84)	3.42 (0.81)	3.52 (0.88)	−0.10	0.11	−0.919	0.359	−0.12
**Connectedness to fellows**	Winter 2021/22	3.11 (1.09)	3.32 (1.08)	2.93 (1.07)	0.39	0.13	3.075	**0.002**	0.37
	Summer 2022	3.27 (1.17)	3.57 (1.12)	2.90 (1.13)	0.66	0.15	4.469	**< 0.001**	0.60

M = mean value. SD = standard deviation. DM = differences of means. SE = standard error of DM. Bold *p*-value = significant after Holm-Bonferroni correction. Cohen's d = effect size of Cohen's d (0.2 ≈ small, 0.5 ≈ medium, and 0.8 ≈ large effect). Distress = Distress Thermometer (range 0–10); Overall symptoms of depression and anxiety = Patient Health Questionnaire-4 (range 0–12); Depression = Patient Health Questionnaire-2 (range 0–6); Anxiety = Generalized Anxiety Disorder-2 (range 0–6); Self-perceived Stress = Perceived-Stress-Scale 4 Items (range 0–12); Change in study motivation (range 1–5); Connectedness to fellows (range 1–5).

**Table 3:** Changes in mental burden and perception of learning environment from winter 2021/22 to summer 2022 in 80 medical students participating in both surveys.

**Variables**	**Survey**	**Longitudinal sample (*n =* 80)**				
		**M (SD)**	**MD (SD)**	***T*** **-value**	***p*** **-value**	**Cohen's** ***d***
**Loneliness**	Winter 2021/22	5.76 (2.02)	0.52 (2.02)	2.326	0.023	0.27
	Summer 2022	5.24 (1.80)				
**Distress**	Winter 2021/22	5.09 (2.65)	−1.39 (3.01)	−4.119	** < 0.001**	−0.54
	Summer 2022	6.48 (2.50)				
**Overall symptoms of depression and anxiety**	Winter 2021/22	4.95 (3.23)	−0.28 (3.50)	−0.704	0.483	−0.09
	Summer 2022	5.23 (3.17)				
**Depression**	Winter 2021/22	2.45 (1.72)	−0.10 (1.89)	−0.474	0.493	−0.06
	Summer 2022	2.55 (1.67)				
**Anxiety**	Winter 2021/22	2.50 (1.75)	−0.18 (1.98)	−0.793	0.430	−0.10
	Summer 2022	2.68 (1.71)				
**Self-perceived Stress**	Winter 2021/22	6.18 (3.54)	0.14 (3.68)	0.487	0.628	0.04
	Summer 2022	6.04 (3.41)				
**Change in study motivation**	Winter 2021/22	3.50 (0.83)	0.10 (0.94)	0.956	0.342	0.13
	Summer 2022	3.40 (0.70)				
**Connectedness to fellows**	Winter 2021/22	2.95 (1.12)	−0.24 (0.98)	−2.159	0.034	−0.20
	Summer 2022	3.19 (1.23)				

M = mean value. SD = standard deviation. MD = mean of differences. Df = degrees of freedom (79). Bold *p*-value = significant after Holm-Bonferroni correction. Cohen's d = effect size of Cohen's d (0.2 ≈ small, 0.5 ≈ medium, and 0.8 ≈ large effect). Loneliness = University of California Los Angeles-3 Items Loneliness Scale (range 3–9). Distress = Distress Thermometer (range 0–10); Overall symptoms of depression and anxiety = Patient Health Questionnaire-4 (range 0–12); Depression = Patient Health Questionnaire-2 (range 0–6); Anxiety = Generalized Anxiety Disorder-2 (range 0–6); Self-perceived Stress = Perceived-Stress-Scale 4 Items (range 0–12); Change in study motivation (range 1–5); Connectedness to fellows (range 1–5).

**Table 4:** Prevalence of loneliness by sociodemographic characteristics and mental burden in 514 medical students in 2022.

		**Total sample**	**Winter 2021/2022**				**Total sample**	**Summer 2022**			
			**Lonely**	**Not lonely**	**χ^2^ (*p*)**	**ϕ**		**Lonely**	**Not lonely**	**χ^2^ (*p*)**	**ϕ**
			**%**	**%**			**%**	**%**	**%**		
**Sociodemographic characteristics**											
**Age**	≤20 years		68.5	31.5	5.124 (0.077)	0.14		51.6	48.4	3.028 (0.220)	0.11
	21–25 years		50.9	49.1				40.6	59.0		
	≥26 years		54.8	45.2				52.6	36.8		
**Gender**	Female		57.6	42.4	1.831 (0.176)	−0.08		47.9	53.1	1.905 (0.168)	−0.09
	Male		48.8	51.2				37.9	62.1		
**Study subject**	Medical		55.9	44.1	0.587 (0.444)	−0.05		45.9	54.1	0.612 (0.434)	−0.05
	Dental		48.1	51.9				37.5	62.5		
**Study year**	Year 1		64.0	36.0	7.879 (0.049)	0.17		40.7	59.3	11.367 (0.010)	0.22
	Year 2		62.7	37.3				63.8	36.2		
	Year 3		46.9	53.1				40.4	59.6		
	Year 4 +		46.8	53.2				36.1	63.9		
**Living situation**	Alone		60.7	39.3	2.653 (0.103)	0.10		50.0	50.0	1.704 (0.192)	−0.09
	Not alone		50.9	49.1				41.4	58.6		
**Relation-ship**	Yes		48.1	52.8	9.280 **(0.002)**	0.18		41.3	58.7	1.405 (0.236)	0.08
	No		65.3	34.7				49.1	50.9		
**Variables of mental burden**											
**Distress**	<Cut-off	35.7	45.5	54.5	5.825 (0.016)	0.14	25.5	33.9	66.1	3.961 (0.047)	0.13
	Cut-off > 4	64.3	60.4	39.6			74.5	48.8	51.2		
**Overall symptoms of depression and anxiety**	Normal		33.8	66.3	28.417 **(< 0.001)**	0.32		30.9	69.1	13.287 **(0.004)**	0.24
	Mild		52.7	47.3				35.1	64.9		
	Moderate		73.1	26.9				53.9	46.1		
	Severe		71.1	28.9				60.5	39.5		
**Depression**	<Cut-off	57.2	42.6	57.4	24.051 **(0.001)**	0.27	48.1	35.1	64.9	8.438 **(0.004)**	0.19
	Cut-off > 2	42.8	71.9	28.1			51.9	54.2	45.8		
**Anxiety**	<Cut-off	55.1	42.3	57.7	23.081 **(< 0.001)**	0.29	33.3	35.8	64.2	6.659 (0.010)	0.17
	Cut-off > 2	44.9	70.9	29.1			66.7	52.8	47.2		

% = percentage of people among category. lonely = students > Cut-off UCLA-3; χ^2^ = chi-square-value. Bold *p*-value = significant after Holm-Bonferroni-correction. ϕ = effect size Phi or Cramer's V (< 0.1 very weak association, 0.1–0.3 weak to moderate association, 0.3–0.5 moderate to strong association, >0.5 strong association). Distress = Distress Thermometer; Overall symptoms of depression and anxiety = Patient Health Questionnaire-4; Depression = Patient Health Questionnaire-2; Anxiety = Generalized Anxiety Disorder-2.

### 3.2 Factors associated with loneliness

We performed binary regression analyses for each measurement time (i.e., model 1 for winter 2021/22 and model 2 for summer 2022). Model 1 explained 22.3% of the variance, whereas Model 2 explained 17.1%. In the winter survey, being single, higher self-perceived stress levels, and decreased study motivation were significantly associated with higher loneliness. The second model demonstrated that other factors became important in the fading COVID-19 pandemic, as lower connectedness with peers was the only factor associated with higher loneliness levels (Table 5).

**Table 5:** Factors associated with loneliness among 514 medical students in the fading COVID-19 pandemic, forced-entry binary logistic regression models.

		**Model 1 in winter 2021/22**				**95% CI**		**Model 2 in summer 2022**				**95% CI**	
		***B***	**SE**	**β**	***p*-value**	**LL**	**UL**	***B***	**SE**	**β**	***p*-value**	**LL**	**UL**
**Sociodemographic characteristics**													
**Age**	Continuous	−0.009	0.051	0.991	0.856	0.896	1.095	0.077	0.055	1.080	0.157	0.971	1.203
**Gender (female** ^*^ **)**	Male	−0.341	0.318	0.711	0.283	0.381	1.325	−0.571	0.344	0.565	0.097	0.288	1.108
**Study Subject (medical** ^*^ **)**	Dental	0.871	0.482	0.419	0.071	0.163	1.077	−0.101	0.520	0.904	0.846	0.326	2.506
**Study Year (Year 1** ^*^ **)**	Year 2	0.117	0.413	1.124	0.778	0.500	2.525	0.901	0.453	2.461	0.047	1.013	5.977
	Year 3	−0.455	0.420	0.635	0.278	0.279	1.445	0.224	0.487	1.251	0.645	0.482	3.250
	Year 4 +	−0.220	0.438	0.802	0.615	0.340	1.892	−0.230	0.509	0.794	0.651	0.293	2.155
**Living Situation (living alone** ^*^ **)**	Not living alone	−0.195	0.287	0.823	0.497	0.469	1.444	−0.087	0.305	0.917	0.776	0.504	1.666
**Relationship (single** ^*^ **)**	Not single	−0.898	0.302	0.407	**0.003**	0.225	0.736	−0.383	0.304	0.682	0.208	0.376	1.238
**Variables of mental burden**													
**Distress (>cut-off** ^*^ **)**	Below cut-off	−0.130	0.331	0.878	0.693	0.459	1.679	−0.190	0.406	0.827	0.639	0.373	1.830
**Depression (>cut-off** ^*^ **)**	Below cut-off	0.324	0.367	1.383	0.377	0.674	2.838	0.307	0.428	1.359	0.473	0.588	3.142
**Anxiety (>cut-off** ^*^ **)**	Below cut-off	0.559	0.343	1.749	0.104	0.892	3.428	−0.01	0.433	0.990	0.981	0.424	2.313
**Self-perceived stress**	Continuous	0.130	0.052	1.139	**0.013**	1.028	1.261	0.113	0.060	1.119	0.058	0.996	1.258
**Change in study motivation**	Continuous	0.532	0.183	1.701	**0.004**	1.188	2.438	−0.139	0.194	0.871	0.476	0.595	1.274
**Connectedness with peers**	Continuous	−0.203	0.134	0.816	0.130	0.627	1.062	−0.452	0.139	0.636	**0.001**	0.484	0.836

n1 = 283, R^2^ = 0.223, Nagelkerkes R^2^ = 0.299. Hosmer-Lemeshow-Test: *p* = 0.136. Omnibus-Test: *p* < 0.001. n2 = 231, R^2^ = 0.171, Nagelkerkes R^2^ = 0.229. Hosmer-Lemeshow-Test: *p* = 0.191. Omnibus-Test: *p* < 0.001. df = degree of freedom (14). B = coefficients of regression, SE = standard error, β = exponential of B. Bold *p*-value = significant after Holm-Bonferroni correction. CI = confidence interval, LL = lower limit, UL = upper limit. Distress = DT score > 4 points, Depression = PHQ-2 > 2 points, Anxiety = GAD-2 > 2 points. (reference^*^) = reference category for categorical variable.

### 3.3 Qualitative data

Over three-quarters of participants responded to the open-ended question regarding current burdensome aspects (82% in winter 2021/2022, 78% in summer 2022). We deducted nine main themes with a response rate of at least 10 percent in at least one survey. In the initial survey, students were most concerned about uncertainties regarding their studies, such as pressure due to learning and possibly insufficient preparation for exams (45 %). This burdened students who participated in the clinical training significantly less than students in the preclinical part of studies (*p* < 0.001). The second most burdensome aspect was a lack of practical experiences and physical attending learning, which was mentioned significantly more often by students from year four and onwards (40%) than lower-year students. Second-year (16%) and year four and higher students (12%) reported greater challenges with the overall study organization communication and infrastructure provided by the faculty.

Participants in the summer 2022 survey were again most frequently concerned about their overall studies (56%). Students in the first year of clinical training (study year 3) were particularly keen on expressing their worries about the lack of practical experience. Overall, students reported more dissatisfaction with the faculty's lack of overall study organization, communication, and infrastructure in the summer survey than in winter (14% vs. 9%). Notably, third-year (28%) and higher students (17 %)—who had already studied before COVID-19—reported burdensome aspects in this category more often than academically younger students. Compared to the winter survey, all burdensome aspects were less frequently reported in summer except for the abovementioned categories. Frequencies of responses did not significantly differ between lonely and not lonely students, aside from the experienced lack of interchange with peers and faculty teachers, which was significantly more often mentioned by the lonely subgroup in the winter survey.

COVID-19-related concerns affected 11% of students in the winter survey and 9% in the summer survey (Table 6). No significant differences in frequencies due to the participant's age, gender, or study subject were observed (data not shown).

**Table 6:** Burdensome aspects of the current study situation in the fading COVID-19 pandemic (open-ended questions, responses of 514 medical students).

		**Total**	**Year 1**	**Year 2**	**Year 3**	**Year 4 +**			**Not lonely**	**Lonel*y***		
		***n*1 =283**	***n*1 = 75**	***n*1 = 67**	***n*1 = 64**	***n*1 = 77**			***n*1 = 127**	***n*1 = 156**		
		***n*2 = 231**	***n*2 = 54**	***n*2 = 58**	***n*2 = 47**	***n*2 = 72**			***n*2 = 127**	***n*2 = 104**		
**Category and subcategory**	**Survey**	**%**	**%**	**%**	**%**	**%**	**χ^2^ (*p*)**	**Cramér's *V***	**%**	**%**	**χ^2^ (*p*)**	**ϕ**
**1. Worries and uncertainties about the overall studies**	Winter 2021/22	45.2	54.7	58.2	26.6	40.3	17.023 **(< 0.001)**	0.25	45.7	44.9	0.018 (0.893)	−0.01
Concerns about possible insufficient preparation for state exams, amount of exams	Summer 2022	55.8	68.5	53.4	48.9	52.8	4.837 (0.184)	0.15	56.7	54.8	0.082 (0.774)	−0.08
**2. Lack of practical experience and physical attendance teaching**	Winter 2021/22	21.9	10.7	16.4	18.8	40.3	22.251 **(< 0.001)**	0.28	19.7	23.7	0.666 (0.415)	0.05
Reduction and cancellation of internships, lack of contact with patients, lack of practical skills	Summer 2022	14.7	7.4	5.2	25.5	20.8	13.033 **(0.005)**	0.24	13.4	16.3	0.399 (0.528)	0.04
**3. Difficulties with self-regulation/self-motivation**	Winter 2021/22	19.1	28.0	20.9	18.8	9.1	8.988 (0.029)	0.18	15.0	22.4	2.534 (0.111)	0.10
Worse work-life-balance, lack of rest and recovery time, missing identification with studies	Summer 2022	11.7	11.1	12.1	17.0	8.3	2.106 (0.551)	0.10	10.2	13.5	0.576 (0.448)	0.05
**4. Burden resulting from online and distance learning**	Winter 2021/22	14.8	9.3	23.9	12.5	14.3	6.428 (0.093)	0.15	10.2	18.6	3.865 (0.049)	0.12
Learning alone/learning at home Lack of quality in remote/online teaching	Summer 2022	5.2	7.4	5.2	10.9	0.0	7.310 (0.063)	0.18	3.9	6.7	0.906 (0.341)	0.06
**5. Self-perceived mental burden**	Winter 2021/22	11.7	14.7	9.0	14.1	9.1	1.956 (0.575)	0.08	9.4	13.5	1.094 (0.296)	0.06
Nervousness, anxiety, insecurity, worries, ruminations, Stress	Summer 2022	7.8	7.4	5.2	12.8	6.9	2.255 (0.521)	0.10	7.9	7.7	0.003 (0.959)	0.00
**6. COVID-19 related worries**	Winter 2021/22	11.3	8.0	10.4	15.6	11.7	2.068 (0.558)	0.09	15.0	8.3	3.066 (0.080)	−0.10
Concern about SARS-CoV-2-infection and contraction, Long-COVID, law restrictions	Summer 2022	8.7	7.4	17.2	0.0	8.3	9.975 (0.019)	0.21	10.2	6.7	0.888 (0.346)	−0.06
**7. Lack of interchange with peers and faculty teachers**	Winter 2021/22	10.6	16.0	14.9	7.8	3.9	7.807 (0.050)	0.17	2.4	17.3	16.500 **(< 0.001)**	0.24
Missing support in conducting doctoral thesis	Summer 2022	2.2	3.7	5.2	0.0	0.0	5.715 (0.126)	0.16	0.0	4.8	6.241 (0.012)	0.16
**8. Lack of social contacts and relationships**	Winter 2021/22	9.9	13.3	9.0	9.4	7.8	1.462 (0.691)	0.07	5.5	13.5	4.963 (0.026)	0.13
Deficiency/lack of friendships with fellow students, missing solidarity and belonging	Summer 2022	1.7	1.9	1.7	2.1	1.4	0.098 (0.992)	0.02	0.8	2.9	1.478 (0.224)	0.08
**9. Lack of overall study organization, communication, and infrastructure provided by the faculty**	Winter 2021/22	8.5	1.3	16.4	4.7	11.7	12.582 **(0.006)**	0.21	6.3	10.3	1.412 (0.235)	0.07
Little to no support from the dean's office, missing concessions by teachers and university officials	Summer 2022	14.3	9.3	5.2	27.7	16.7	12.247 (0.007)	0.23	16.5	11.5	1.166 (0.280)	−0.07

*n* = number of participants in t1 (n1) and t2 (n2). % = percentage. Two categories were deleted (responses < 10 % during both surveys). χ^2^ = chi-square value. Bold *p*-value = significant after Holm-Bonferroni-correction. ϕ = effect size Phi (< 0.1 very weak association, 0.1–0.3 weak to moderate association, 0.3–0.5 moderate to strong association, >0.5 strong association). Effect sizes for Cramér's V are identical.

## 4 Discussion

This study aimed to fill the gap of lacking follow-up data on the mental burden of medical students during the fading COVID-19 pandemic in Germany. Overall, medical students in our study experienced high remaining levels of distress, depression, and anxiety during the fading COVID-19 pandemic, aligning with findings from other research on the outgoing pandemic in China (Cheng et al., 2023). In more detail, participants expressed clinically relevant levels of depressive symptoms more frequently than the general population [19.1% (Cohen, 1988), 26.9% (de Sousa et al., 2021)], as well as medical students before [pooled prevalence: 27.2% (Rotenstein et al., 2016)] and during COVID-19 [pooled prevalence: 37.9% (Jia et al., 2022)]. Likewise, the prevalence of anxiety symptoms was higher compared to other medical students before COVID-19 [pooled prevalence: 33.8% (Quek et al., 2019)] and during COVID-19 [pooled prevalence: 33.7% (Jia et al., 2022)]. Comparable, perceived stress levels in both the winter and the summer survey of our study were higher than in the German norm population before COVID-19 [*M* = 4.8 (4.0) (Klein et al., 2016)] and German medical and dental students in 2020 [*M* = 5.6 (3.1) (Guse et al., 2021b)].

### 4.1 Phenomenon of loneliness in medical students

Our study's particular focus was the assessment of loneliness among medical students. About half of our participants experienced loneliness during our research. The frequency of loneliness among medical students was higher compared to other studies [42.5 % (Pop et al., 2022) and 21.0% (Keiner et al., 2023)] during COVID-19. Similarly, previous studies measuring loneliness with the same instrument as we reported lower means of loneliness among medical students before COVID-19 [*M* = 5.5 (1.9) (Alkureishi et al., 2022)], *M* = 3.5 (2.6) (Werner et al., 2021)], and in 2020 [*M* = 5.2 (2.2) (Werner et al., 2021)] than we did (*M* = 5.8 [2.0] in winter, *M* = 5.2 [1.9] in summer). Students who participated in both surveys reported lower loneliness levels in the summer survey, presumably due to the loosening of COVID-19 governmental and academic restrictions (HRK, 2022). This assumption is supported by the fact that explicitly pandemic-related burdensome aspects, such as burdens from online and distance teaching, lack of interchange with peers and faculty teachers, and lack of social contacts and relationships, are less frequently addressed in the summer than in the winter survey. Ultimately, all participants' connectedness to fellows was slightly higher in the summer survey. Surprisingly, most self-reported burdensome factors did not significantly differ between lonely and not-lonely participants, deriving that burdensome factors did apply the same way for all students.

We thereby conclude an improvement in social isolation and, thus, loneliness among medical students during the fading COVID-19 pandemic during our study.

### 4.2 Understanding differences in medical students' well-being

Even though age did not play a significant role in our analyses in explaining differences in loneliness amongst medical students, so did participants' study year. Strikingly, first-year students experienced the steepest decrease in frequency of loneliness (−23.3 %) from winter 2021/22 to summer 2022. In contrast, second-year students expressed roughly the same share in both surveys. Students in clinical training have experienced at least one semester of regular study conditions, including first-year welcome festivities and peer tutoring programs from other medical students before COVID-19. This might have ensured a relevant resource in the following years of COVID-19 restrictions. On the other hand, most second-year students were affected by COVID-19 during their final school exam phase in 2020. Further, they did not participate in regular students' lives at the beginning of their studies, likely resulting in a lack of peer-group bonding. Moreover, most second-year students prepared for the first part of the medical licensing examination in the summer of 2022. Similarly, preparation made them face more worries and uncertainties about possible examinational failure than clinical students. Simultaneously, first-year students might have benefited most from reinstalled academic programs and normalization in the summer of 2022 (HRK, 2022). The qualitative part of our surveys further underlines that preclinical students felt most burdened by exam preparation and the amount of matter. In contrast, students near graduation felt most insecure about their possible lack of skills. In addition to students' dissatisfaction with the faculty's study organization, communication, and infrastructure, plausible cause for rises in overall distress during summer 2022 exists. Qualitative research explained similar findings regarding pre-gradational students (van den Broek et al., 2020) and overall medical students (Chew-Graham et al., 2003). To tackle these challenges, a combined longitudinal study program might help untangle exam pressure in the preclinical section. Additionally, adding more guided and supervised teaching of practical skills in clinical curricula is advised.

Another factor contributing to the difference in reported loneliness between students in preclinical vs. clinical training might be an overhang of symptoms of mental burden after experiencing stress. Previous research during a SARS outbreak in Hong Kong 2003 has gained similar insights, concluding that stress among affected healthcare workers does not decrease as restrictions do (McAlonan et al., 2007). Challenges in social identity formation (SIF) might also help develop an understanding of this data, as identification with groups and adopting their rules and values is crucial to a person's social identity (Tajfel, 1979).

Social identity formation is largely considered an essential part of medical education (Misra et al., 2022). However, the challenges of COVID-19, such as strict social distancing, prohibited many forms of regular social interactions. Transitional processes among medical students add another critical factor in understanding study year differences. Transitional processes in adolescents and young adults have previously been identified as a risk factor for loneliness for young students (Diehl et al., 2018). Both time points of arriving and departing medical school put a risk to students' well-being. As for intervention, we suggest this burden can be approached by strengthening peer support through small buddy-peer programs and initiating extracurricular social support groups in compliance with health regulations.

### 4.3 Relevance for medical education

Lastly, results from binary regression reveal the most special insights: previous studies identified young age or living alone (Hysing et al., 2020; Diehl et al., 2018), depression (Werner et al., 2021), or anxiety (Diao et al., 2023) as predictors of loneliness among students. However, the abovementioned factors were not associated with higher levels of loneliness in our sample. Furthermore, female gender did not predict loneliness, which contradicts most other research (Hysing et al., 2020; Werner et al., 2021; Labrague et al., 2021). In general, specific gender-related effects in mental health problems between men and women occur. For instance, depression is more prevalent and more frequently diagnosed in women than in men (Parker and Brotchie, 2010; Karger, 2014). On the other side, gender-related effects also concern men, as single men are a high-risk group for loneliness, and similarly, living alone is a significant risk factor for loneliness among male students (Hysing et al., 2020). We emphasize that loneliness is a mental health concern that affects all students, regardless of gender. Thus, support needs to address all students. Our results from binary regressions further underline that, similar to previous findings, particular interest should be paid to single students and students who feel highly stressed (Zhang et al., 2021). Interestingly, factors associated with loneliness differed in the course of our study, as connectedness to peers emerged as a risk factor in the fading COVID-19 pandemic in summer 2022. Hence, we underline that the abovementioned strategies to strengthen connectedness in medical students (small-buddy programs, social support groups) might help to reduce loneliness. Low-threshold counseling services at academic institutions, as well as structural mentoring programs for students who encounter mental health problems early on in their studies, could be a means to tackle the overall high mental burden in medical students, including loneliness (Wasson et al., 2016). The need for counseling offers is further stressed as clinical students reported a lack of overall support by faculty in our study. This is particularly important as fear of stigmatization is a known barrier to students seeking help for mental health concerns, as demonstrated by previous research (Chew-Graham et al., 2003).

Thus, the climate in academia should create an inclusive and non-judgmental environment, enabling students to reach out for help if needed. Further, support strategies should be individualized, as evidence suggests that coping strategies among medical students vary considerably with regard to age, study year, and gender (Fitzgibbon and Murphy, 2023). In conclusion, support from academic institutions is strongly advised to help relieve mental health burdens among medical students.

### 4.4 Limitations

When interpreting the study results, certain limitations should be kept in mind. First, our study samples are relatively modest in size (response rate around 20 %). This was most likely caused by the voluntariness of participating, contributing to a particular selection bias. Students affected by mental health concerns might be more likely to participate in studies addressing mental health issues, whereas unaffected people might tend to overlook such research. Given this reasoning, a plausible overrepresentation of burdened participants might have occurred. Though this response rate is similar to other studies performed during COVID-19 (Weber et al., 2022), we like to stress that our findings have limited generalizability to different student populations and the general population. Future data collection should aim to minimize this bias, for instance by considering alternative recruiting methods, or by assessing reasons of non-respondence. Similarly crucial, Germany is considered a Western, educated, industrialized, rich, and democratic (WEIRD) country. This means our findings may not be transferable to other regions of the world. Worldwide, SARS-CoV-2 has spread differently, thus resulting in different dynamics of contagion and overall impact. Comparisons between countries are limited to specific time points, which may differ in terms of infection rates and death toll. For instance, China experienced some COVID-19 relief at the end of 2020, while a second unprecedented pandemic outbreak hit Germany (World Health Organization, 2024). In the absence of data dating back to pre-pandemic times, comparisons to a baseline level of mental burden among medical students are limited.

## 5 Conclusion

Depressive symptoms, anxiety levels, and perceived stress among German medical students persisted at high levels during the fading COVID-19 pandemic, while general distress even increased. However, levels of loneliness decreased in our study, likely due to the overall reduction of COVID-19 regulations, marking one of the first studies to observe this development during the post-pandemic phase. Levels of distress, depression, and anxiety are higher among lonely students than unbothered students in the declining, confirming a continuing observation.

In contrast to many other studies, we could not identify gender and age as factors associated with loneliness among medical students, indicating loneliness is a global phenomenon possibly affecting all medical students, regardless of gender or age. Consequently, action must be taken to develop inclusive support strategies. Risk factors for loneliness in medical students differed in the fading pandemic, with students disconnected from their fellows at higher risk. Thus, special attention should be paid to revitalizing and initiating peer-related bonding programs. Differences in loneliness between study years underline the unique needs of preclinical students, which academic institutions should address. Qualitative analysis revealed that specific pandemic-related burdens did decrease over time. In contrast, worries and uncertainties about the overall studies, including pressure due to exam preparation, remained the most burdensome concerns for students. This highlights the importance of addressing academic pressures alongside mental health concerns in medical students, with a particular focus on reducing stress through structural changes in medical curricula.

## Data Availability

The datasets presented in this article are not readily available because ethical approval was obtained for researchers' use only. Requests to access the datasets should be directed to marvik.leich@med.uni-greifswald.de.
