# Supplementary material for: Loneliness and mental burden among German medical students during the fading COVID-19 pandemic: a mixed-methods approach
Source: Front Psychol. 2025 Mar 25;16:1526960. doi: 10.3389/fpsyg.2025.1526960 (PMC11975857; doi:10.3389/fpsyg.2025.1526960)
Supplement: Supplementary file 1 [file Table_1.docx]

| **Supplement 1: Differences in levels of mental burden among medical students by study year in the fading COVID-19 pandemic** | | | | | | | | | | | |
| --- | --- | --- | --- | --- | --- | --- | --- | --- | --- | --- | --- |
|  |  | **Total** | **Year 1** | **Year 2** | **Year 3** | **Year 4 +** |  |  |  |  |  |
|  |  | n1 = 283 | n1 = 75 | n1 = 57 | n1 = 64 | n1 = 77 |  |  |  |  |  |
|  |  | n2 = 231 | n2 = 54 | n2 = 58 | n2 = 47 | n2 = 72 |  |  |  |  |  |
|  | **survey** | **M (SD)** | **M (SD)** | **M (SD)** | **M (SD)** | **M (SD)** | **SQ** | **MS** | **F-value** | **p-value*** | **η²** |
| **Loneliness** | winter 2021/22 | **5.77** (2.02) | **6.03** (2.01) | **6.04** (1.97) | **5.64** (2.00) | **5.40** (2.05) | 21.460 | 7.153 | 1.776 | .152 | 0.019 |
|  | summer 2022 | **5.22** (1.90) | **5.20** (2.12) | **5.78** (1.62) | **5.04** (1.84) | **4.90** (1.89) | 26.660 | 8.887 | 2.525 | .058 | 0.032 |
| **Distress** | winter 2021/22 | **5.43** (2.65) | **5.55** (2.65) | **5.48** (2.81) | **6.08** (2.40) | **4.73** (2.60) | 66.080 | 22.027 | 3.202 | .024 | 0.033 |
|  | summer 2022 | **6.42** (2.70) | **7.28** (2.44) | **7.38** (2.20) | **6.43** (2.31) | **5.00** (2.89) | 238.291 | 79.430 | 12.591 | **<.001*** | 0.143 |
| **Overall symptoms of depression and anxiety** | winter 2021/22 | **4.98** (3.23) | **5.61** (3.36) | **5.16** (3.35) | **5.39** (2.98) | **3.86** (2.96) | 140.229 | 46.743 | 4.645 | **.003*** | 0.048 |
|  | summer 2022 | **5.42** (3.32) | **6.96** (3.40) | **6.26** (3.10) | **5.28** (2.86) | **3.67** (2.93) | 391.653 | 130.551 | 13.807 | **<.001*** | 0.154 |
| **Depression** | winter 2021/22 | **2.44** (1.71) | **2.77** (1.73) | **2.49** (1.82) | **2.69** (1.52) | **1.87** (1.63) | 37.444 | 12.481 | 4.428 | **.005*** | 0.045 |
|  | summer 2022 | **2.64** (1.81) | **3.46** (1.83) | **3.07** (1.66) | **2.57** (1.67) | **1.71** (1.61) | 109.940 | 36.647 | 12.887 | **<.001*** | 0.146 |
| **Anxiety** | winter 2021/22 | **2.54** (1.80) | **2.84** (1.87) | **2.67** (1.84) | **2.70** (1.71) | **1.99** (1.70) | 33.158 | 11.053 | 3.491 | .016 | 0.036 |
|  | summer 2022 | **2.78** (1.80) | **3.50** (1.84) | **3.19** (1.71) | **2.70** (1.53) | **1.96** (1.66) | 86.622 | 28.874 | 10.097 | **<.001*** | 0.118 |
| **Self-perceived Stress** | winter 2021/22 | **6.30** (3.63) | **7.07** (3.49) | **6.90** (3.84) | **6.09** (3.74) | **5.19** (3.25) | 164.616 | 54.872 | 4.310 | **.005*** | 0.044 |
|  | summer 2022 | **6.42** (3.49) | **7.74** (3.61) | **6.97** (3.40) | **6.40** (3.50) | **5.00** (3.00) | 256.648 | 85.549 | 7.611 | **<.001*** | 0.091 |
| **Change in study  motivation** | winter 2021/22 | **3.51** (0.84) | **3.48** (0.84) | **3.52** (0.84) | **3.41** (0.94) | **3.62** (0.75) | 1.755 | 0.585 | 0.829 | .479 | 0.009 |
|  | summer 2022 | **3.46** (0.80) | **3.13** (0.62) | **3.41** (0.96) | **3.55** (0.86) | **3.69** (0.80) | 10.381 | 3.460 | 5.200 | **.002*** | 0.064 |
| **Connectedness to  fellows** | winter 2021/22 | **3.11** (1.09) | **3.03** (1.13) | **3.03** (1.17) | **3.22** (1.06) | **3.16** (1.00) | 1.865 | 0.622 | 0.524 | .666 | 0.006 |
|  | summer 2022 | **3.27** (1.17) | **3.61** (1.22)* | **3.02** (1.08) | **3.49** (1.12) | **3.07** (1.16) | 15.146 | 50.449 | 3.843 | .010 | 0.048 |
| Annotations: n = frequencies, t1 = survey 1, t2 = survey 2. M = mean value, SD = standard deviation. SQ = sum of squares, df = degree of freedom. MS = mean squares. η² = effect size eta², f = effect size f. p* < 0.05 Loneliness = University of California Los Angeles-3 Items Loneliness Scale (range 3-9). Distress = Distress Thermometer (range 0–10); Overall symptoms of depression and anxiety = Patient Health Questionnaire-4 (range 0-12); Depression = Patient Health Questionnaire-2 (range 0–6); Anxiety = Generalized Anxiety Disorder-2 (range 0-6); Self-perceived Stress = Perceived-Stress-Scale 4 Items (range 0 - 12); Change in study motivation (range 1-5); Connectedness to fellows (range 1-5). | | | | | | | | | | | |
